# Knowledge, Attitude, and Practice of Livestock Owners and Livestock Assistants towards African Trypanosomiasis Control in The Gambia

**DOI:** 10.1155/2022/3379804

**Published:** 2022-01-24

**Authors:** Alpha Kargbo, Edrisa Jawo, Amien Isaac Amoutchi, Herve Koua, Rex Kuye, Zainabou Dabre, Abdoulie Bojang, Rafael F. C. Vieira

**Affiliations:** ^1^WASCAL-Graduate Research Program in Climate Change and Biodiversity, Universite Felix Houphouet-Boigny, BP V34, Abidjan, France; ^2^Department of Physical and Natural Sciences, University of the Gambia, Brikama Campus, P. O Box 3530, Serrekunda, Gambia; ^3^Laboratoire de Zoologie et Biologie Animale, Université de Cocody, 22 BP 582 Abidjan 22, France; ^4^Department Public Health and Environmental, School of Medicine and Allied Health Science, University of the Gambia, Brikama Campus, P. O Box 3530, Serrekunda, Gambia; ^5^Remot Sensing Department, Ahmadu Bello University, Zaria, Kaduna, Nigeria; ^6^Vector-Borne Diseases Laboratory, Department of Veterinary Medicine, Universidade Federal do Paraná, Curitiba, PR, Brazil; ^7^Global One Health Initiative (GOHi), The Ohio State University, Columbus, OH, USA

## Abstract

**Background:**

In Africa, it has been estimated that 50 million cattle and 70 million sheep and goats are at risk of animal African trypanosomiasis, and three million cattle die annually.

**Methods:**

This study was conducted in all the regions of The Gambia except Kombo Saint Mary Island (Banjul). Structured questionnaires were administered to 440 randomly selected livestock owners and 23 livestock assistants, and 7 focus group discussions were held for both livestock owners and livestock assistants. The data were analyzed mainly using descriptive statistics and content analysis methods.

**Results:**

A total of 94.5% and 75% of livestock owners reported having seen tsetse and horse flies, respectively, while 100% of livestock assistants reported having seen tsetse flies. Forty-seven percent of the livestock owners indicated a positive attitude toward control measures, while 42% of them had no idea how to control tsetse flies. On the other hand, 57% of livestock assistants believe that tsetse and horse flies are the main reasons why AAT is still in their community. There was a statistically significant difference between all the respondents' characteristics and the practices done by livestock owners to prevent AAT vectors from biting their animals.

**Conclusion:**

This study shows that trypanosomiasis is still a major problem for livestock health and production in The Gambia, and it requires disease and vector control.

## 1. Background

African animal trypanosomiasis (AAT) is a neglected zoonotic parasitic disease caused by protozoans of the genus *Trypanosoma* [1, 2], and it causes important economic losses of approximately 4.5 billion US dollars per year [3] as a result of direct (mortality, production losses, costs of prophylactic, and curative trypanocidal drugs) and indirect losses due to crop spoilage and agricultural worker involvement (deficiency of animal protein diets) [4, 5]. Tsetse flies of the genus *Glossina* and horse flies of the genus *Tabanidae* are the main vectors for trypanosomes [2, 6]. In humans, the disease is known as sleeping sickness or human African trypanosomiasis (HAT), caused by *Trypanosoma brucei gambiensis* and *Trypanosoma brucei rhodesiense* [6, 7]. In livestock, the disease is known as “nagana” or AAT and is widespread across sub-Saharan Africa [2, 8].

In Africa, it has been estimated that 50 million cattle and 70 million sheep and goats are at risk of AAT, and three million cattle die annually [9]. Livestock rearing in Africa has mainly affected the health, livelihoods, and environment of African people. In many regions of the continent, the demand for livestock products has surpassed domestic production. This demand is expected to be fueled further by population growth, urbanization, and income growth in all African nations [10–12]. *Trypanosoma* control and prevention can only be attained through vector control by regular dipping of animals in chemicals since vaccine production has not yet been achieved [12]. Moreover, Diminazene aceturate and Isometamidium chloride have been used to cure trypanosomiasis for the past three decades, and there is a possibility that there could be some cases of resistance to these drugs [11, 12].

In The Gambia, AAT affects livestock productivity, with equids being very vulnerable [13–15]. The West Africa Livestock Innovation Center (WALIC), through the Ministry of Agriculture and the Horse and Donkey Trust Fund (HDTF), is the two institutions making some effort to control trypanosomiasis in The Gambia. Since there is no available vaccine for trypanosomiasis, WALIC has attempted to select and genetically improve indigenous trypan-tolerant breeds such as N'dama and Zebu cattle to control the incidence of trypanosomiasis in the country and neighboring countries [15]. Zebu cattle are more productive in terms of meat and milk production, although more vulnerable to AAT than N'dama cattle, and they cannot survive for long in both the Central River Region (CRR) and Lower River Region (LRR), regions highly infested with tsetse flies [15]. HDTF has been more active in curing trypanosomiasis-related cases in livestock animals, especially in equids across the country [16]. Currently, there is no official AAT vector control program in The Gambia. Accordingly, the study is aimed at evaluating the knowledge, attitude, and practice (KAP) of livestock owners and livestock assistants toward the AAT control in The Gambia.

## 2. Materials and Methods

### 2.1. Study Area

**Figure 1:**
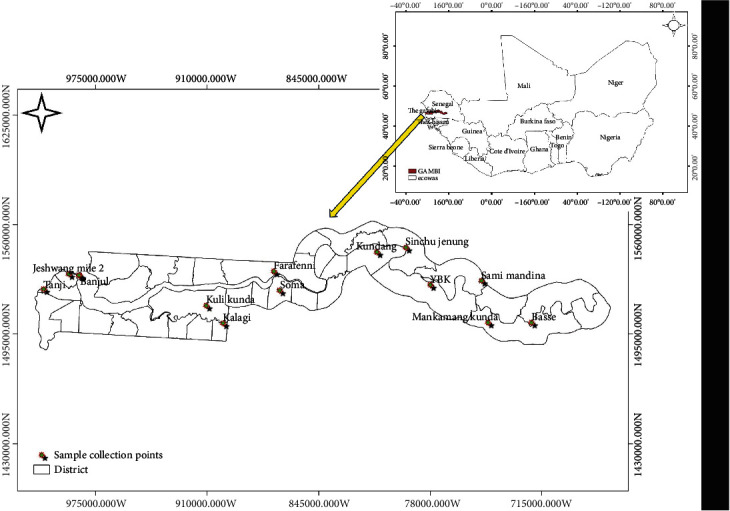
Map of The Gambia showing its position in West Africa and the location of the present study. The figure was generated and modified using ArcGIS 9.

The Gambia is the smallest country in mainland Africa and is located in West Africa. It consists of seven administrative regions and one independent city called Banjul (Figure 1). Among the districts selected for this study, the Kanifing Municipal Council and Banjul were the only districts located in the urban area. Participants were chosen randomly from sixteen districts out of 46 districts in The Gambia.

### 2.2. Study Design and Sampling Technique

A cross-sectional survey was conducted from October 2020 to January 2021 to assess and compare the KAP regarding AAT in The Gambia using a standardized questionnaire [11, 16] and focus group discussion (FGD).

The sample size was calculated by Yamane [17] based on an estimated number of 724,952 livestock owners in The Gambia [18] with a level of precision of 0.05%. Thus, the minimum sample size required to detect a difference with a 95% confidence level of 5% was estimated as *n* = 440 participants. Two districts and one village per district were randomly selected for each region in The Gambia. A list of all livestock owners was obtained from the veterinary service office in the districts under study. The names of the 440 livestock owners (351 males and 89 females) evaluated were randomly selected from the lists provided by the livestock officer serving in each region. Additionally, 23 livestock assistants were also interviewed. A total of seven FGD were evaluated, five for livestock owners and two for livestock assistants.

### 2.3. Inclusion and Exclusion Criteria

All inhabitants > 18 years, residing in the specified region for >36 months, and able to communicate were considered eligible for the study.

### 2.4. Method of Data Collection

A standardized questionnaire was developed based on a previous study [12, 19]. The questionnaire comprised 17 questions that were divided into four categories: (1) sociodemographic characteristics; (2) knowledge on the vectors, clinical signs, transmission, and control of trypanosomiasis; (3) economic impact of trypanosomiasis; (4) attitude towards prevention of trypanosomes; and (5) practices regarding trypanosome prevention.

### 2.5. Data Analysis

The data were entered into the Microsoft Excel software, and either Chi-square or Fisher's exact test was used to assess differences in the proportions of individual variables using Statistical Package for Social Sciences (SPSS) version 25 (IBM Corp., Armonk, NY, USA), and binary logistic regression was used to determine the association between the explanatory variable, and knowledge of vectors was implemented using the R 3.6.3 software. The results were considered significant when *P* ≤ 0.05. The correlation coefficient was used to describe the correlation between knowledge, attitude, and practice regarding AAT.

## 3. Results

### 3.1. Livestock Owners and Livestock Assistants' Profile

A total of 351/440 (80%) livestock owners were male between 40 and 49 years old (132/351; 37.6%), while 21/23 (91.3%) livestock assistants were male between 18 and 29 years old (7/21; 30.4%). Tables 1 and 2 summarize the profile for both groups.

The majority of livestock owners raised only cattle (195/440; 44.3%) and cattle and small ruminants (147/440; 33.4%), followed by cattle and equids (47/440; 10.7%) and cattle, small, ruminants and equids (51/440; 11.6%). Herd sizes varied from 1 to 50 (225/440; 55.1%), 51 to 100 (138/440; 31.3%), 101 to 150 (53/440; 12.0%), and >150 (24/440; 5.45%).

### 3.2. Knowledge of Vectors and Trypanosomiasis

A total of 416/440 (94.5%) livestock owners reported the occurrence of tsetse flies, 330/440 (75%) reported horse flies, and all livestock assistants reported the occurrence of AAT vectors. Livestock owners reported that AAT vectors are well known in their local language as follows: tsetse flies are known as “Kosse” in Wolof, “Joloh” in Mandinka, and “Kaifeh” in Fula, while horse flies are also known as “Kusigi” in Wolof, “Sue Joloh” in Mandinka, and “Daaso” in Fula. Furthermore, AAT is known the Wolofs as “Toi” in horses and “Darsoh” in other livestock, “Koojah” in Mandinka, and “Darsoh” in Fula. Respondents' knowledge of tsetse flies was significantly influenced by all their demographic variables. The results from the binary logistic regressions showed a positive relationship between ethnic group (*P* ≤ 0.01), gender (*P* = 0.015), age (*P* = 0.035), occupation (*P* = 0.04), and qualification (*P* ≤ 0.001) and knowledge of tsetse flies. The respondents' knowledge of the occurrence of horse flies was influenced by their age (*P* = 0.001), ethnic group (*P* ≤ 0.001), and qualification (*P* ≤ 0.001) (Table 3).

In FGD, participants claimed the occurrence of tsetse flies in the bushes near their community, in animals' stalls, and generally when livestock was drinking at the well. A total of 411/440 (93.4%) and 325/440 (81.36%) livestock owners reported the occurrence of tsetse and horse flies in their communities, respectively. Additionally, 358/440 (81%) livestock owners reported knowing that tsetse flies transmit *Trypanosoma*, while 286/440 (65%) reported that their animals had been previously diagnosed with trypanosomiasis. Although 410/440 (93.18%) livestock owners had access to veterinary practitioners, only 110/440 (25%) reported previously collected blood samples to diagnose *Trypanosoma* spp. in their animals. When livestock assistants were interviewed, 13/23 (57%) reported that they conducted a microscopy laboratory diagnosis of *Trypasonoma* spp.

From the 440 livestock owners, the clinical signs of AAT described were sluggishness (410; 93%), loss of weight (330; 75%), low productivity (59; 13%), intermittent fever (120; 27%), and death (425; 97%). Livestock assistants reported having good knowledge of the clinical signs of AAT, including loss of weight (12; 52%), anemia (11; 48%), pale mucous membrane (7; 30%), abortion (3; 13%), nervous disorder (8; 35%), rough coat (9; 39%), weakness (9; 39%), eye discharge (8; 35%), fever (8; 35%), death (1; 4%), elasticity of skin (9; 2%), and swollen vulva or tsetse (4; 17%). There was a statistically significant difference between respondents' characteristics and whether they knew the disease called trypanosomiasis (Table 4).

### 3.3. Attitude to Trypanosomiasis

A total of 251/440 (57%) livestock owners reported that the presence of tsetse flies is the reason why AAT is still prevalent in their community, while 101/440 (23%) reported that it is due to the nearby river and 9/440 (2%) to the presence of wild animals in their forest. However, all livestock assistants reported that AAT is prevalent in The Gambia because of the presence of the main vectors and lack of official control measures to mitigate the disease in the country.

### 3.4. Practice to Prevent Insect Biting Livestock Animals

A total of 209/440 (47%) livestock owners reported the use of chemicals to control tsetse/horse flies from biting their animals, 9/440 (2%) used resistant breeds, 6/440 (1%) cleared bushes around the compound, 13/440 (3%) reported the use of all of the above control measures, and 22/440 (5%) reported that AAT was impossible to control. Moreover, 194/440 (44%) livestock owners reported that they had no idea of how to control tsetse/horse flies. Sociodemographic variables and practices of livestock owners to control vectors from biting their animals in The Gambia are summarized in Table 5.

### 3.5. Impact of Trypanosomiasis on Livestock Owners

A total of 370/440 (84%) livestock owners reported that the cost to treat one infected animal with trypanosomiasis is US$ 2, and if they are to use prophylaxis, the cost is usually US$ 4 per cattle. This was also confirmed by the FGDs for both respondent groups.

## 4. Discussion

In the present study, 94.5% and 75% of the livestock owners had knowledge about tsetse and horse flies, respectively, and 100% livestock assistants reported the occurrence of these vectors in their communities and stations, in agreement with previous studies [12, 19–21]. Tsetse flies are known as “Joloh” in Mandinka, “Kosse” in Wolof, and “Kaifeh” in Fula, while horse flies are known as “sue joloh” in Mandinka, “kusigi” in Wolof, and “Daaso” in Fula. During the FGD, livestock owners were well informed about the presence of biting flies in their communities. Most of the respondents described differentiating tsetses from house flies as annoying insects that usually greatly disturb animals. A livestock owner in one of the FGD reported that “Whenever we see an animal is running without anything pursuing it or making a groaning noise most of the times is either tsetse or other flies which usually bites them.” However, most of the livestock owners (409/440, 93%) also reported that these vectors are larger and have a longer mouth part than house flies and can even bite human beings. This classification made it difficult for them to be able to differentiate between tsetse or house flies and/or other similar insects, although 75% of livestock owners describe horse flies as “flies with big head and some have colored green or red eyes,” similar to previous studies conducted in Tanzania [12] and Uganda [22]. In those studies, the members of the community classified these vectors as “annoying insects which bites both humans their animals” [12, 22]. Herein, livestock owners and assistants also indicated that these vectors are highly prevalent in The Gambia, and they are well known to be the vector of AAT and HAT in the country.

In the present study, AAT was well known by livestock owners and assistants. During FGD, livestock owners described AAT as a disease similar to malaria in livestock (81%), while others described it as being a disease that is caused by hunger (19%). According to livestock owners and assistants, AAT is one of the main hindrances to livestock production and advancement in The Gambia. This fact might be due to the pathogenicity seen in cattle and the understanding that bush, water, and forests, which are favorable habitants for *Trypanosoma* spp. infection by these vectors, especially in non-*N'dama* breeds of cattle. Similarly, our data are in agreement with previous studies that ranked AAT as the most vital limitation to cattle production in East Africa [21, 23, 24], and previous studies have also shown that livestock farmers have strongly reported that AAT is the major problem for livestock productivity and agricultural development in Ethiopia [24, 25] and in Kenya [21].

Herein, 93% of livestock owners and 100% of livestock assistants showed a comparatively good understanding of the clinical signs of bovine trypanosomiasis. Our data agree with the findings previously reported in other countries in Africa [24, 26, 27]. However, 93% of livestock owners reported that they had access to livestock assistants' officers, and most of them (66%) reported that their animals had been previously infected by *Trypanosoma* spp. Furthermore, one of the FGDs with livestock assistants reported that the Department of Livestock Services employs only three qualified veterinary doctors, with approximately 80 livestock assistants serving in all administrative regions in The Gambia. This lack of more qualified veterinarians usually hampered veterinary service delivery in the country. However, 75% of livestock assistants reported that AAT diagnosis is performed based on judgmental and not clinical factors. It was also revealed in FGD that there is only one functional government-owned veterinary laboratory situated at Abuko (urban area) in The Gambia, which makes conducting laboratory analysis difficult in the rural area, even though WALI and HDTF try to complement the effort of The Gambia government by providing veterinary services to infected animals within CRR, LRR, and WD.

Sex (*P* = 0.008), ethnic group (*P* ≤ 0.001), region (*P* ≤ 0.001), and education level (*P* ≤ 0.001) were among the main factors that significantly influenced livestock owners' knowledge of AAT in The Gambia (Table 4). Livestock owners and livestock assistants showed decent attitudes toward AAT. This study agrees with a similar finding in Senegal [28], who also showed that farmers had positive attitudes toward tsetse flies and trypanosomiasis.

Herein, livestock assistants reported that they mainly use Diminazene aceturate, trypamidium, and Isometamidium chloride (Veridium) to treat sick animals suspected of AAT, with trypamidium (Samorin) being usually used as prophylaxis (data not shown). According to one FGD, livestock owners occasionally use the abovementioned drugs to treat their animals suspected of AAT even though they do not have any formal training on drug administration. They do so simply because they try to avoid the cost of paying extra for the treatment of their sick animals if they are to call a livestock assistant. However, the high frequency of trypanocidal application coupled with the report of self-preparation and injection of the drugs by some livestock owners shows that there is a high risk of possible development of trypanocidal resistance in The Gambia. The frequent act of treating unconfirmed trypanosomiasis cases with trypanocidal certainly leads to the development resistance of *Trypanosoma* species [21, 24, 28]. On the other hand, 47% of livestock owners reported that they applied chemicals to their animals to prevent tsetse bites. The present study agrees with a previous study performed in Tanzania [19], who reported that farmers practice dipping animals in chemicals to prevent tsetse flies from biting animals in communities neighboring Serengeti National Park Tanzania. “In the evenings, we light a fire near the animal stalls to keep biting insects at bay, and during the day, we dress our animals' limbs to keep insects at bay, especially if they have a wound on that limb” (FGD). However, 42% of livestock owners reported that they had no idea on what to do to prevent these vectors from biting their animals. Most livestock owners reported that “Whenever we suspect our animals of having trypanosomiasis, we feed them with Mahogany leaves combined with salt or Detarium sengalense” (FGD).

The only government-led program to control trypanosomiasis in The Gambia is financing the activities of the WALI. It is also worth noting that a livestock assistant noted in another FDG that “Through WALI, local breeds are known as N'dama cattle and Jallonke sheep which are resistant to trypanosomiasis are usually crossed breed with other breeds and the animals with higher resistance ability are usually sold to farmers living in the high trypanosomiasis prevalent zones in The Gambia” (FGD). On the other hand, livestock assistants usually advise farmers to keep animals in secure houses, use insect dipping, grazing animals in open fields rather than bush areas, and light a fire at night to prevent vectors from biting livestock (FGDs). Respondents' characteristics and the practices done by livestock owners to prevent vectors from biting their animals were statistically associated: sex (*P* = 0.013), age (*P* = 0.014), ethnic group (*P* ≤ 0.001), region (*P* ≤ 0.001), occupation (*P* = 0.001), and education level (*P* < 0.001).

This study shows that livestock farmers in all regions and of all ages are aware of AAT and how to control the disease in their animals. This finding is similar to previous studies by Barrow et al. [29], who also reported that there was a higher significance of age and gender in practices that control and prevent schistosomiasis in The Gambia. Finally, 84% of livestock owners reported expending US$ 2 and 16% expending US$ 3 to treat one of their cattle suspected of having trypanosomiasis depending on the severity of the animal's ailment. The burden of treatment for this disease further helps to impoverish livestock owners in The Gambia. This finding corroborates with previous studies in Kenya [7, 21], which reported that AAT has significantly affected the settlements and economic development of farmers in most countries in Africa, particularly those south of the Sahara Desert, in which The Gambia is definitely not an exception.

## 5. Conclusion

The survey conducted on the knowledge, attitude, and practice of livestock owners and livestock assistants on the occurrence of trypanosomiasis in The Gambia has provided us with important information on the status of AAT, and the KAP of livestock owners and livestock assistants affects disease control. Livestock owners and livestock assistants also recognized that tsetses are associated with trypanosomiasis and had good knowledge of the clinical signs of AAT and its impact on the livelihood and well-being of cattle as well as on the owners. As a result, we urge that more research to be conducted in The Gambia to establish the efficiency of routinely used trypanocidal drugs. Finally, we urge that The Gambia government and other stakeholders invest in the development of DLS staff's human resource capacity, as well as the future development of all veterinary laboratories in the country, to ensure the appropriate diagnosis of AAT.

## Figures and Tables

**Table 1:** Sociodemographic profile of livestock owners from The Gambia, 2021.

Characteristics	Frequency (*n* = 440)	Percentage (%)
Sex		
Male	351	80
Female	89	20
Age		
0-29	57	13
30-39	91	20.7
40-49	132	30
50-59	64	14.5
60-69	60	13.6
>70	36	8.2
Qualification		
Primary	111	25.2
Secondary	49	11.1
Tertiary	9	2.3
Quranic education	124	28.2
None	147	33.2
Main occupation		
Herdsmen	220	50
Crop farming	19	4
Livestock rearing	124	28
Crop and livestock farming	4	1
Petty trading	73	17
Region		
WCR	15	3.4
LRR	75	17
NBR	75	17.3
CRR-S	56	12.8
CRR-N	111	25.2
URR	108	24.3

WCR: West Coast Region; LRR: Lower River Region; NBR: North Bank Region; CRR-S: Central River Region-South; CRR-N: Central River Region-North; URR: Upper River Region.

**Table 2:** Demographic profile of livestock assistants from The Gambia, 2021.

Characteristics	Frequency (*n* = 23)	Percentage (%)
Sex		
Male	21	91
Female	2	9
Age		
18-29	7	30.4
30-39	6	26.1
40-49	6	26.1
50-59	3	13
>60	1	4.3
Region		
KMC	5	22
WCR	3	13
URR	3	13
LRR	3	13
NBR	3	13
CRR-N	3	13
CRR-S	3	13

WCR: West Coast Region; LRR: Lower River Region; NBR: North Bank Region; CRR-S: Central River Region-South; CRR-N: Central River Region-North; URR: Upper River Region.

**Table 3:** Association between sociodemographic variables and knowledge of African animal trypanosomiasis vectors in The Gambia, 2021.

	Knowledge of tsetse flies		Knowledge of horse flies	
	Coefficient standard error	*P*	Coefficient standard error	*P*
Ethnic group	0.509	0.01^∗^	0.343	≤0.001^∗∗^
	(0.199)		(0.105)	
Gender	1.222	0.015^∗^	0.187	0.55
	(0.507)		(0.311)	
Age	-0.355	0.035^∗^	0.266	≤0.001^∗∗^
	(0.168)		(0.079)	
Occupation	-0.421	0.04^∗^	-0.104	0.20
	(0.204)		(0.082)	
Qualification	-0.402	0.001^∗∗^	-0.346	≤0.001^∗∗^
	(0.146)		(0.073)	

^∗^ *P* < 0.05; ^∗∗^*P* < 0.01.

**Table 4:** Characteristics of livestock owners and knowledge of African animal trypanosomiasis in The Gambia, 2021.

Characteristics	Do you know the disease called trypanosomiasis (*n* = 440)	Frequency (%)	*X* ^2^	*P* value
	Yes			
Sex			15.74	0.008^∗∗^
Male	261	59		
Female	56	13		
Age			48.168	0.004^∗∗^
0-29	39	9		
30-39	64	15		
40-49	102	23		
50-59	42	10		
60-69	44	10		
>70	26	6		
Ethnic group			50.185	**≤**0.001^∗∗^
Mandinka	147	33		
Fula	99	23		
Jola	16	4		
Wolof	53	12		
Others	2	0.5		
Education			74.361	**≤**0.001^∗∗^
Primary	74	17		
Secondary	38	9		
Tertiary	8	2		
Madarasa	101	23		
None	96	22		
Region			171.25	**≤**0.001^∗∗^
WCR	15	3		
LRR	57	13		
NBR	61	14		
CRR-S	40	9		
CRR-N	80	18		
URR	64	15		
Occupation			73.248	**≤**0.001^∗∗^
Herdsmen	162	37		
Crop farming	14	3		
Livestock farming	83	19		
Crop and livestock farming	3	0.7		
Others	55	13		

Others include fisher forks, housewives, craft workers, and petty traders. WCR: West Coast Region; LRR: Lower River Region; NBR: North Bank Region; CRR-S: Central River Region-South; CRR-N: Central River Region-North; URR: Upper River Region. ^∗^*P* < 0.05; ^∗∗^*P* < 0.01.

**(a):** 

Characteristics	How to control tsetse flies							
	Use of chemical *N* = 206	Use of improved breeds *N* = 9	Clearing of bushes *N* = 6	Impossible to control tsetse *N* = 22	All of the above *N* = 13	I do not know *N* = 184	Total *N* = 440	*P* value
Sex								0.013^∗^
Male	173 (39.5%)	9 (2.1%)	6 (1.4%)	17 (3.9%)	12 (2.7%)	133 (30.3%)	351	
Female	32 (7.3%)	0 (0%)	0 (0%)	5 (1.1%)	1 (0.2%)	51 (11.6%)	89	
Age								0.014^∗^
0-29	21 (4.9%)	0 (0%)	1 (0.2%)	2 (0.5%)	5 (1.1%)	28 (6.4%)	57	
30-39	45 (10%)	2 (0.5%)	0 (0%)	4 (0.9%)	0 (0%)	40 (9.1%)	90	
40-49	66 (15%)	2 (0.5%)	2 (0.5%)	4 (0.9%)	3 (0.7%)	55 (12.5%)	132	
50-59	36 (8.2%)	0 (0%)	2 (0.5%)	2 (0.5%)	1 (0.2%)	23 (5.2%)	64	
60-69	27 (6.2%)	2 (0.5%)	0 (0%)	8 (1.6%)	3 (0.7%)	20 (4.6%)	60	
>70	11 (2.5%)	3 (0.7%)	1 (0.2%)	2 (0.5%)	1 (0.2%)	18 (4%)	36	
Ethnic group								<0.001^∗∗^
Mandinka	103 (23.5%)	4 (0.9%)	0 (0%)	6 (1.4%)	1 (0.2%)	75 (17%)	189	
Fula	50 (11.4%)	5 (1.1%)	1 (0.2%)	9 (2.1%)	12 (2.7%)	72 (16.4%)	148	
Jola	10 (2.3%)	0 (0%)	2 (0.5%)	3 (0.7%)	0 (0%)	14 (3.2%)	29	
Wolof	39 (8.9%)	0 (0%)	3 (0.7%)	4 (0.9%)	0 (0%)	22 (5%)	68	
Others	4 (0.9%)	0 (0%)	0 (0%)	0 (0%)	0 (0%)	1 (0.2%)	5	

**(b):** 

Characteristics	How to control tsetse flies							
	Use of chemical *N* = 206	Use of improved breeds *N* = 9	Clearing of bushes *N* = 6	Impossible to control tsetse *N* = 22	All of the above *N* = 13	I do not know *N* = 184	Total *N* = 440	*P* value
Education								≤0.001^∗∗^
Primary	27 (6.2%)	6 (1.4%)	0 (0%)	13 (3%)	12 (2.7%)	53 (12.1%)	111	
Secondary	28 (6.4%)	2 (0.5%)	0 (0%)	1 (0.23)	0 (0%)	18 (4.1%)	49	
Tertiary	6 (1.4%)	0 (0%)	1 (0.2%)	0 (0%)	1 (0.2%)	2 (0.5%)	10	
Madarasa	77 (17.5%)	0 (0%)	0 (0%)	2 (0.5%)	0 (0%)	45 (10.3%)	124	
None	68 (15.5%)	1 (0.2%)	5 (1.1%)	6 (7%)	0 (7%)	66 (15%)	146	
Region								≤0.001^∗∗^
WCR	1 (0.2%)	5 (1.1%)	0 (0%)	2 (0.5%)	7 (1.6%)	0 (0%)	15	
LRR	35 (8%)	1 (0.2%)	1 (0.2%)	4 (0.9%)	0 (0%)	34 (7.8%)	75	
NBR	32 (7.3%)	0 (0%)	4 (0.9%)	4 (0.9%)	0 (0%)	36 (8.2%)	76	
CRR-S	33 (7.5%)	0 (0%)	0 (0%)	1 (0.2%)	0 (0%)	22 (5%)	56	
CRR-N	54 (12.3%)	1 (0.2%)	1 (0.2%)	10 (2.3%)	6 (1.4%)	39 (8.9%)	111	
URR	51 (11.6%)	2 (05%)	0 (0%)	1 (0.2%)	0 (0%)	53 (12.1%)	107	
Occupation								≤0.001^∗∗^
Herdsmen	106 (24.1%)	6 (1.4%)	2 (0.5%)	13 (3%)	10 (2.3%)	83 (19%)	219	
Crop farming	6 (1.4%)	1 (0.2%)	1 (0.2%)	0 (0%)	3 (0%)	8 (1.8%)	19	
Livestock	50 (11.4%)	1 (0.2%)	3 (0.7%)	7 (1.6%)	0 (0%)	63 (14.4%)	124	
Crop and livestock farming	2 (0.5%)	1 (0.2%)	0 (0%)	0 (0%)	0 (0%)	1 (0.2%)	4	
Others	42 (9.5%)	0 (0%)	0 (0%)	2 (0.5%)	0 (0%)	29 (6.6%)	73	

^∗^ *P* < 0.05; ^∗∗^*P* < 0.01.

## Data Availability

The data will be made available upon request.

## Abbreviations

AAT: Animal African trypanosomiasis
HAT: Human African trypanosomiasis
KAP: Knowledge attitude practice
FGD: Focus group discussion
WALIC: West Africa Livestock Innovative Center
HDTF: Horse and Donkey Trust Fund
CRR-S: Central River Region-South
CRR-N: Central River Region-North
WCR: West Coast Region
LRR: Lower River Region
URR: Upper River Region
KMC: Kanifing Municipal Council.
